# A reproducible workflow for isolating and characterizing bacterial endophytes, pathogens, and saprophytic colonizers from tomato fruits

**DOI:** 10.1016/j.mex.2026.103889

**Published:** 2026-03-27

**Authors:** Abraham Goodness Ogofure, Ezekiel Green, Etinosa Ogbomoede Igbinosa

**Affiliations:** a Department of Biotechnology and Food-Technology, Faculty of Science, University of Johannesburg, South Africa; b Molecular Pathogenic and Molecular Epidemiology Research Group (MPMERG), Department of Biotechnology and Food-Technology, Faculty of Science, University of Johannesburg, South Africa

**Keywords:** Tomato fruits, Endophytes, Bacterial soft rot, Plant pathogens, Saprophytes, *Pectobacterium carotovorum*

## Abstract

A reproducible workflow is presented that integrates fruit‑health stratification, stringent surface sterilization, culture‑based isolation, molecular identification, enzyme phenotyping, and a low‑injury needle‑transfer pathogenicity assay to isolate, classify and functionally characterize bacterial endophytes, saprophytic colonizers and pathogens associated with postharvest tomato (*Solanum lycopersicum*) fruits. The method is designed to distinguish ecological guilds (endophytes vs soft‑rot pathogens vs saprophytes) rather than simply list “bacteria present”, and can be implemented in standard microbiology laboratories without specialized equipment. Tomato fruits were stratified by fruit-health status and surface‑sterilized to distinguish internal endophytes from epiphytic and saprophytic surface‑associated communities. All bacterial isolates were cultured on tryptone soya agar, purified, assigned to an ecological niche (healthy or spoiled/diseased fruits), and tested for *in planta* pathogenicity on tomato fruits.

All the isolates were identified using biochemical and 16S rRNA gene sequencing, while preliminary phenotypic screening was used to quantify cell wall‑degrading activities relevant to soft‑rot. The workflow yielded 14 characterized bacterial isolates spanning three ecological groups (non‑pathogenic endophytic *Bacillus* species, soft‑rot‑inducing Enterobacterales, and saprophytic colonizers), with ecological niche separation statistically supported by Fisher’s exact test (*p* < 0.001). The method can be adapted to other fruit or vegetable systems to link bacterial community composition with plant health outcomes.

• Provides a low-injury and contamination-reduced approach for fruit pathogenicity assays based on a needle‑transfer inoculation technique adapted for routine microbiology laboratories.
• Enables the functional differentiation of endophytic, saprophytic, and pathogenic bacterial isolates relevant to fruits and vegetables through combined ecological sources, in planta pathogenicity, and enzyme phenotyping.
• The approach is adaptable to multiple fruit and vegetable host crops in a resource-limited and efficient laboratory setting.

## Graphical abstract

**Figure:**
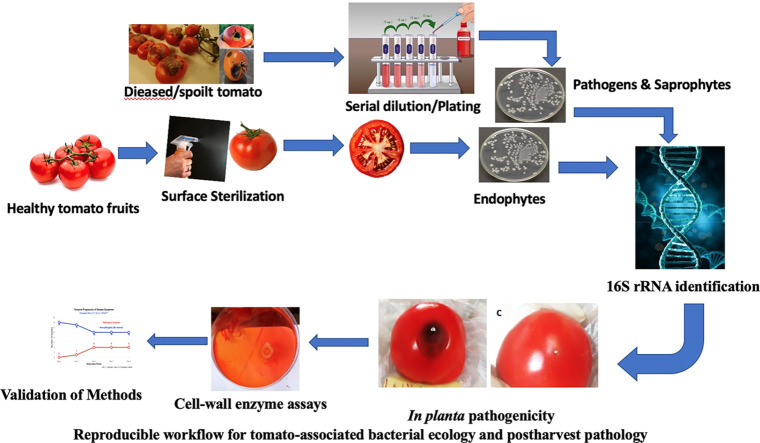

**Table:** Specifications table.

Subject area	Immunology and Microbiology
**More specific subject area**	*Plant Pathogenic Microbiology*
**Name of your method**	*Needle-Transfer-Inoculation Technique on Tomato Fruits*
**Name and reference of original method**	*Orzaez, D., S. Mirabel, W.H. Wieland, and A. Granell, Agroinjection of tomato fruits. A tool for rapid functional analysis of transgenes directly in fruit. Plant Physiol, 2006. 140(1): p. 3–11.*
**Resource availability**	Data are available in Figshare with doi: 10.6084/m9.figshare.30997672 and accession numbers PQ605804.1, PQ605803.1, PQ605802.1, PQ605801.1, MK814569.1, MK814568.1, MK813904.1, MK814570.1, PQ605810.1, PQ605809.1, PQ605808.1, PQ605807.1, PQ605806.1, PQ605805.1 are also available on the NCBI website.

## Background

Postharvest soft-rot of fruits (such as tomato (*Solanum lycopersicum*)) represents a significant food security and safety concern, particularly in low- and middle-income countries. This is usually driven by several factors, including high pathogen pressure, weak handling practices, and inadequate cold-chain infrastructure, leading to significant economic losses and other safety concerns [1,2]. Most fruits with postharvest rots are encountered in our local markets, where they are sold at very low or giveaway prices. These market environments provide a realistic interface between microbial ecology and consumer exposure, as fruits at different stages of integrity might harbour diverse bacterial communities. Based on the foregoing, isolates recovered from broken or soft-rotted tomato fruits may belong to distinct ecological guilds, such as saprophytes, endophytes, or opportunistic/true pathogens. Thus, distinguishing these functional roles would be crucial for understanding postharvest disease dynamics and consumer-associated risks.

Pathogenicity testing on healthy tomato fruits is a critical step in this process, as it is required to differentiate isolates into distinct ecological guilds. However, the conventional fruit inoculation technique reported in the literature, particularly the cork borer method involving excision of fruit tissue and reinsertion after inoculation, presents several practical limitations [[3], [4], [5]]. Amongst these are the facts that large tissue wounds can induce physiological stress (unrelated to microbial virulence) on a fruit as delicate as a tomato. Moreover, the technique, which is time-consuming (when processing multiple isolates), requires considerable technical skill and may increase the likelihood of cross-contamination due to repeated tissue handling.

These limitations pose serious challenges for laboratories with high sample throughput, limited resources, and personnel with varying levels of technical expertise. Therefore, there was a need to develop a simplified yet reproducible inoculation strategy that minimizes fruit injury, reduces contamination risk, and enables efficient evaluation of multiple bacterial isolates on a single fruit.

The modified needle-inoculation method described herein addresses these constraints by introducing bacterial cells into the fruit tissue via a controlled micro-wound rather than a large excision site (cork borer). This approach mimics natural entry points for infection while reducing excessive tissue damage associated with the cork borer technique, and saving precious time and energy. More importantly, this methodological approach was designed to be implemented in a microbiological laboratory without specialized equipment and can be easily adapted for other fruits or vegetables to link bacterial community composition with plant health outcomes. This protocol is suitable for studies investigating postharvest microbial ecology, pathogenicity screening and functional differentiation of fruit-associated microbiota in plant pathology and applied food safety research contexts.

The workflow described here, therefore, goes beyond simple isolation and identification. It (i) stratifies fruits by health status, (ii) applies rigorous surface sterilization to define “true” endophytes, (iii) uses a needle‑transfer inoculation method that minimizes fruit injury and cross‑contamination, and (iv) combines pathogenicity outcomes with basic enzyme phenotyping and 16S rRNA sequencing to assign isolates to ecological guilds. This integrated sequence of steps, together with full public deposition of sequences and an explicitly resource‑adapted protocol, represents the main methodological novelty of the work. In practical terms, the method allows laboratories to move from “a list of culturable bacteria” to an ecologically meaningful classification of fruit‑associated isolates into endophytes, pathogens, and saprophytes, thereby providing a tractable tool for postharvest disease surveillance and risk assessment in settings where advanced infrastructure is limited.

## Method details

### Sample collection and preparation

A total of 120 fresh, healthy-looking tomato fruits (*Solanum lycopersicum*) devoid of visible lesions, bruises, or decay, and 120 soft-rotten fruits were purchased from five randomly selected open markets in Benin City, Nigeria. Samples were collected between January and March 2019. Healthy fruits were processed for endophyte recovery, while soft-rotten/diseased fruits were processed for recovery of bacterial saprophytes and pathogens. All samples were transported to the laboratory under aseptic conditions and processed within 4 h. of collection. The healthy tomato fruits were surface-sterilized by sequential immersion in 95% ethanol, followed by 1.5% sodium hypochlorite, and rinsed multiple times with sterile distilled water to eliminate epiphytic microorganisms [6,7]. Sterilized fruits were aseptically sectioned and macerated for isolation of internal bacterial populations. For the soft-rotten/diseased fruit samples, tissue from the margins of the healthy and rotten zones was aseptically macerated and plated on TSA under the same conditions to recover saprophytic and pathogenic bacteria [8]. Plates with growth were sub-cultured repeatedly to obtain pure cultures, and all isolates were preserved on TSA slants at 4 °C for downstream analyses. All isolation procedures (homogenization, serial dilution, and plating) were performed in triplicate (*n = 3* independent biological replicates) for each fruit‑health category (healthy vs diseased) to ensure reproducibility of presence/absence patterns.

### Isolation and cultivation of bacterial isolates from healthy and diseased tomato fruits

Healthy tomato fruits obtained from the market (to intentionally reflect real-world post-harvest conditions and microbial communities relevant to consumer exposure and encountered in the food supply chain) were processed within 4 h. of collection to minimize post-harvest microbial proliferation. Bacterial endophytes were isolated from the healthy fruits (without any sign/symptom of rot/spoilage) after a series of 5 washes with distilled water to remove any debris. The fruits were surface-sterilized by immersion in 70% ethanol for 5 min., followed by two distilled-water rinses, then another round of sterilization with 1.5% sodium hypochlorite solution for 7 min., and another series of 5 rinses with distilled water (to eliminate residual disinfectants and epiphytes, if present). Finally, the last rinse water (100 µL) was plated as the control, and if the culture (last rinse water) yielded no growth after incubation, then it is certain that only true endophytes were isolated from the sterilized fruits [6,7,9], confirming the successful removal of epiphytes and environmental isolates. The sterilized fruits were then sectioned aseptically using sterile carpel and macerated (under a laminar airflow chamber) before being spread-plated (100 µL) on tryptone soya agar (TSA) plates. Meanwhile, for spoiled/soft-rotten fruits, aliquots (100 µL) of homogenized fruit tissues were serially diluted and plated onto TSA plates. This difference in pre‑processing (surface‑sterilized whole healthy fruits versus lesion‑margin tissue from soft‑rotten fruits) was intentional and reflects the ecological roles being investigated: internal endophytes in apparently healthy fruits versus saprophytic and pathogenic colonizers of rotting tissue, rather than a quantitative comparison of total bacterial loads between matrices. The culture plates for spoiled fruits were incubated at 28 ± 2 °C for 24–48 h, while the culture for healthy fruit samples (endophytes) was incubated at the same temperature for 24–96 h and observed daily for growth. Plates with growth were sub-cultured repeatedly to obtain pure cultures, and all Isolates were preserved on TSA slants at 4 °C for downstream analyses. All isolations were performed in triplicate to ensure reproducibility.

### Phenotypic characterization and culture standardization

Pure isolates were characterized based on colony morphology, Gram staining, cell shape, and standard biochemical tests (catalase, oxidase, motility and basic sugar reactions) using conventional protocols. For downstream assays, overnight cultures in Tryptone Soy Broth were adjusted to a turbidity equivalent to a 0.5 McFarland standard (≈1.5 × 10⁸ CFU/mL) and kept in the refrigerator for further molecular analysis.

### DNA extraction, 16S rRNA amplification and sequencing

Genomic DNA was prepared from 24‑h broth cultures using a boiling lysis protocol. Briefly, 2 mL of culture was centrifuged at 10,000 × *g* for 5 min; the pellet was resuspended in 200 µL sterile distilled water, vortexed, heated at 100 °C for 15 min, and then centrifuged at 10,000 × *g* for 2 min; the supernatant served as the DNA template. Nearly full‑length 16S rRNA genes were amplified using primers 27F (5′‑AGAGTTTGATCMTGGCTCAG‑3′) and 1540R (5′‑TACGGYTACCTTGTTACGACT‑3′) in a 50 µL PCR mixture containing 10 µL of 5 × GoTaq reaction buffer (final 1 ×), 1 µL of dNTP mix at 10 mM each (final 0.25 mM of each dNTP), 3 µL of 25 mM MgCl₂ (final 1.5–1.8 mM), 1 µL (10 pmol; final ∼0.2 µM) of each primer, 0.3 U of GoTaq DNA polymerase (Promega), and 8 µL of template DNA with nuclease‑free water added to 50 µL. Cycling conditions were: initial denaturation at 94 °C for 5 min; 30 cycles of 94 °C for 30 s, 50 °C for 60 s and 72 °C for 90 s; final extension at 72 °C for 10 min. Amplicon was confirmed on 1.5% agarose gels stained with ethidium bromide [8,10]. PCR products were purified by ethanol–sodium acetate precipitation and resuspended in sterile water before bidirectional sequencing on an ABI 3130xl Genetic Analyzer using BigDye terminator chemistry. Resulting sequences were trimmed and compared against the NCBI 16S rRNA database using BLASTn, and species‑level identities were assigned based on ≥99% similarity. All sequences were deposited in GenBank, and accession numbers are provided in Table 1.

**Table 1:** Molecular identification and ecological classification of bacterial isolates from tomato fruits.

Ecological Group	Bacterial Species	Strain	Fruit Source	GenBank Accession
Endophytes	Bacillus xiamenensis	AMPEHREG1	Healthy	PQ605804.1
	Bacillus pumilus	Tomato2	Healthy	PQ605803.1
	Bacillus safensis	Tomato1	Healthy	PQ605802.1
	Bacillus australimaris	Veadams1	Healthy	PQ605801.1
Pathogens	Salmonella enterica	Provy2019	Diseased	MK814569.1
	P. carotovorum	PENA2019	Diseased	MK814568.1
	Serratia marcescens	AOE2019	Diseased	MK813904.1
	Leclercia adecarboxylata	AVH2019	Diseased	MK814570.1
Saprophytes	Enterobacter cloacae	TE3	Diseased	PQ605810.1
	Kluyvera ascorbata	TE4	Diseased	PQ605809.1
	Citrobacter freundii	TE2	Diseased	PQ605808.1
	Providencia vermicola	UNIBEN20	Diseased	PQ605807.1
	Providencia rettgeri	MCB02	Diseased	PQ605806.1
	Proteus mirabilis	AMPEHREG2	Diseased	PQ605805.1

### Phylogenetic and ecological classification

Partial 16S rRNA gene sequences of bacterial isolates were retrieved from the NCBI GenBank database using their respective accession numbers and compiled into a single FASTA file. Sequence headers were standardized to contain isolate identifiers only, ensuring compatibility with downstream phylogenetic visualization tools. Multiple sequence alignment was performed using MAFFT with the automatic strategy selection option, which optimizes alignment parameters based on sequence length and similarity [11]. To improve alignment quality and remove poorly aligned or ambiguously positioned regions, the resulting alignment was trimmed using trimAl with the automated1 algorithm. A phylogenetic tree was inferred from the trimmed alignment using the FastTree algorithm under a nucleotide substitution model, which efficiently estimates approximately maximum-likelihood phylogenies for large or moderately sized datasets. The resulting tree was exported in Newick format and used for downstream visualization. Phylogenetic tree visualization, annotation, and formatting were performed using the Interactive Tree of Life (iTOL) web platform, enabling clear representation of taxonomic relationships among the isolates [11]. All analyses were conducted using command-line tools in a Unix-based environment to ensure reproducibility. Isolates were then classified ecologically as endophytes (recovered from fruits, which have been surface‑sterilized and are non‑pathogenic *in planta*), primary pathogens (isolated from diseased fruits and also positive for soft rot on healthy fruits), or saprophytes (from diseased fruits but non‑pathogenic *in planta*). It should be noted that while multilocus sequence analysis (MLSA) using housekeeping genes usually provides higher taxonomic resolution for bacterial isolates of closely related taxa, 16S rRNA sequencing was employed as the methodology of choice in the study due to its wide acceptance in environmental and plant microbiome studies. We acknowledge this limitation of single-gene identification, and future studies will incorporate MLST to refine species-level associations.

### *In planta* pathogenicity assays on healthy tomato fruits

Pathogenicity test was adopted to determine whether the isolated microbes from diseased tomato fruits were responsible for the spoilage or disease condition when tested on healthy fruits. The suspected causal pathogen implicated in bacterial soft rot was isolated from a diseased fruit and grown in pure culture. The isolated bacterial pathogen(s) were then used to inoculate healthy tomato fruits. The newly inoculated healthy tomato fruits were assessed for the development of soft rot disease symptoms. In cases where symptoms occurred, the causal pathogen(s) were isolated and cultured in fresh media and characterized. The identity of the newly characterized pathogen(s) was compared with that of the original pathogen introduced into the healthy host. When the same organism was consistently re‑isolated from symptomatic tissue, this was interpreted as fulfilling the classical Koch’s postulates for that isolate, whereas the presence or absence of symptoms per se was used to score its pathogenicity (ability to cause disease). However, in cases where disease symptoms did not develop, it was considered a negative test for pathogenicity. Based on the foregoing, a preliminary pathogenicity screening was conducted using a needle-transfer inoculation technique previously described in the literature [12], with modifications. Fresh, symptom-free, and healthy tomato (*Solanum lycopersicum*) fruits were sequentially surface-sterilized by immersion in absolute ethanol (95%) for 5 min. and then, sodium hypochlorite (1.5% v/v) for 7 min. These immersions were followed by multiple rinses with sterile distilled water and allowed to air-dry under aseptic conditions (Laminar flow hood). Bacterial cultures were grown for 24 h and standardized to a 0.5 McFarland turbidity corresponding to 1.5 × 10^8^ CFU/mL. A total of ten bacterial isolates were selected for pathogenicity testing based on their preliminary biochemical characteristics and more so the fact that they were isolated from the spoiled/broken tomato fruits obtained. Sterile sewing pins/needles were immersed in a standardized suspension of bacterial cells to allow adherence to the needle surface. Each pin was then aseptically inserted into or used to puncture the tomato fruit pericarp to a depth of approximately 5–8 mm to create a defined point of inoculation, and this procedure was repeated for replicate fruit samples. The sewing needles were left *in planta* (in the fruits) throughout the incubation period to clearly mark the inoculation site and minimize exposure of the wound to external microorganisms. Control fruits were pierced with needles dipped in sterile distilled water only, and each bacterial isolate was tested in replicate fruits. The inoculated fruits were incubated at ambient laboratory temperature (25 ± 2 °C) under humid conditions and monitored daily from Day 0 (day of inoculation) to Day 4 (day of termination), for development of water‑soaked lesions, tissue maceration, or soft rot around the needle entry point. The bacterial isolates were classified as pathogenic if reproducible lesions were developed at the inoculation site in all replicates, whereas the absence of visible symptoms was scored as a negative reaction. Therefore, the appearance of visible lesions, tissue softening, or maceration around the point of inoculation was recorded as a positive pathogenicity response, while the absence of visible symptoms was recorded as a negative response. No additional manipulation or removal of the inoculation needles was performed during the observation period. ​Three replicate fruits were inoculated with each isolate; lesion diameter was measured daily using a calibrated meter rule, and, for isolates that produced lesions, bacteria were re‑isolated from symptomatic tissue and compared with the original inoculum to provide experimental support consistent with Koch’s postulates.

### Qualitative assessment of cell wall–degrading enzyme activity

Cell wall–degrading enzyme activity of the bacterial isolates was evaluated using qualitative plate-based assays for cellulolytic and pectinolytic activity [13,14] with modification. All bacterial isolates were first cultured on tryptone soya agar (TSA) for 24 h to obtain actively growing colonies. Briefly, a 6 mm sterile Cork borer was used to remove agar plugs containing actively growing bacterial culture from the TSA plates. For cellulolytic activity, the agar plugs were placed onto the already freshly prepared carboxymethyl cellulose (CMC) agar plates, while pectinolytic activity was assessed by placing agar plugs onto formulated pectinolytic agar. All assays were performed in replicates and all inoculated plates were incubated at room temperature for 24 h. Following incubation, cellulase detection was performed by flooding CMC plates with 1% (w/v) Congo red solution, rinsed with 1 M NaCl, and examined for clear halos around the agar plugs against a red background. For pectinase detection, pectin agar plates were flooded with 1% (w/v) iodine solution and observed for decolorized zones around the plugs. The appearance of a distinct clear zone was recorded as a positive reaction for the respective enzyme, whereas the absence of a halo was scored as negative. For enzyme‑positive isolates, the diameter of the clear zone (including the plug) was measured in millimetres using a ruler. Zone diameters were measured in three replicate plates, and the mean ± standard deviation was calculated. A zone diameter >10 mm was considered strong activity, 5–10 mm moderate, and <5 mm weak.

### Data analysis and visualization

All datasets generated in this study were analyzed using descriptive and comparative statistical approaches to ensure transparent presentation of experimental outcomes. Categorical data, including isolate distribution, pathogenicity screening results, and enzyme activity profiles, were summarized as frequencies and proportions. Associations between isolate source and fruit health status were evaluated using Fisher’s exact test, while relationships between isolate identity and lesion development were assessed using chi-square analysis, with statistical significance evaluated at *p* < 0.05. Phylogenetic relationships were inferred from partial 16S rRNA gene sequences and visualized as a distance-based tree to illustrate genetic relatedness among isolates. Data visualization was performed using R (version 4.5.1), employing packages including *ggplot2* and *ComplexHeatmap* to generate high-resolution figures [6,7,9]. All plots were standardized for axis scaling, colour schemes, and labelling to ensure clarity and reproducibility, and no data transformations were applied beyond those required for visualization.

### Method validation

In total, 120 healthy‑appearing fruits and 120 soft‑rotten fruits were sampled from five independent markets, and the isolation workflow was applied in triplicate for each fruit‑health category. The same four *Bacillus* species were recovered exclusively from healthy‑fruit replicates, whereas the same ten Enterobacterales species were recovered exclusively from diseased‑fruit replicates across all three biological replicates. The method or approach we employed in the study provided comprehensive molecular and phenotypic characterization of bacterial diversity in tomato (*Solanum lycopersicum*) fruits from Nigerian markets. Through systematic isolation, 16S rRNA gene sequencing, and pathogenicity screening, we characterized 14 bacterial species representing three distinct ecological groups: (i) four endophytic *Bacillus* species isolated from healthy fruits that exhibited no pathogenic potential, (ii) four pathogenic species from diseased fruits capable of causing soft rot disease, and (iii) six saprophytic colonizers isolated from diseased fruits but lacking pathogenic activity when tested *in planta* in healthy tomato fruits.

The molecular identification and ecological classification of bacterial isolates from tomato fruits is shown in Table 1. The molecular identification of bacterial isolates from diseased and healthy tomato fruits documents the presence of *Bacillus xiamenensis, Bacillus pumilus, Bacillus safensis, Bacillus australimaris, Salmonella enterica, P. carotovorum, Serratia marcescens, Leclercia adecarboxylata, Enterobacter cloacae, Kluyvera ascorbata, Citrobacter freundii, Providencia vermicola, Providencia rettgeri* and *Proteus mirabilis*. The summary of molecular identity and ecological grouping of all 14 representative isolates recovered from tomato fruits (Table 1) documents that soft-rotten tomatoes contained predominantly saprophytic and pathogenic bacterial isolates. However, fewer isolates were obtained from healthy tomato fruits. The GenBank accessions of the isolates are also displayed in the last column (Table 1). Furthermore, the result shows that four *Bacillus* spp. were exclusively recovered from surface‑sterilised healthy fruits, whereas ten Enterobacterales species were isolated from diseased fruits and partitioned into four primary soft‑rot pathogens and six non‑pathogenic saprophytes. The table serves as a reference dataset linking molecular identity to ecological origin.

**Fig. 1:**
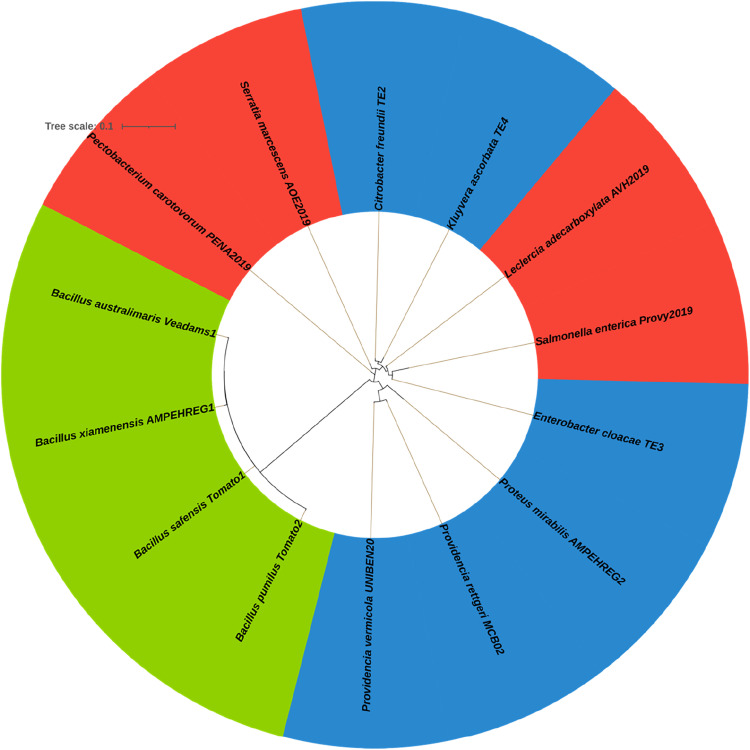
Phylogenetic relatedness of bacterial isolates from diseased and healthy tomato fruits.

The phylogenetic relatedness of bacterial isolates from tomato fruits is shown in Fig. 1. The circular phylogenetic tree illustrates the genetic relationships among bacterial isolates recovered from tomato fruits based on partial 16S rRNA gene sequences. All sequences were aligned and used to construct a nucleotide-based phylogeny, with branch lengths reflecting genetic divergence amongst the bacterial isolates from healthy and soft-rotten tomato fruits. The phylogenetic tree generated using interactive tree of life highlights the taxonomic clustering of isolates into distinct genera, including *Bacillus, Pectobacterium, Proteus, Citrobacter, Serratia, Salmonella, Providencia, Kluyvera, Enterobacter*, and *Leclercia*. All isolates identified as *Bacillus* species form a coherent cluster distinct from Enterobacterales-associated taxa and they are represented using the green label (Fig. 1). Bootstrap support values ranged from 75–100% for major clades, indicating robust phylogenetic relationships. The phylogenetic structure provides a sequence-based framework for contextualizing the ecological distribution and pathogenicity-related datasets presented in subsequent figures.

**Fig. 2:**
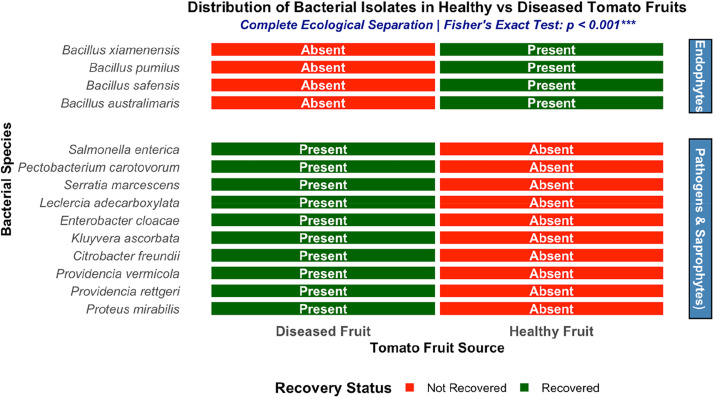
Distribution of bacterial isolates recovered from healthy and diseased tomato fruits.

The distribution of bacterial isolates from diseased and healthy tomato fruits is shown in Fig. 2. The summary of the distribution of bacterial isolates recovered from soft-rotten tomato fruits as well as the fresh/healthy fruits (a reflection of the conditions of the tomato fruits at the point of isolation) showed that soft-rotten/diseased tomatoes had all 10 bacterial isolates obtained in our study. Only four bacterial isolates of the *Bacillus* genus (*B. xiamenensis, B. pumilus, B. safensis, and B. australimaris*) were recovered from apparently healthy fruits. These isolates, based on their isolation methodology were basically classified as putative endophytes. All four *Bacillus* species were recovered from healthy fruits, whereas all Enterobacterales species were detected only in diseased fruits, supporting their classification as pathogens or saprophytes rather than opportunistic or true endophytes. Fisher's exact test indicated a highly significant difference in the distribution of bacterial isolates between diseased and healthy tomato fruits (*p* < 0.001), demonstrating complete ecological niche separation with no shared species between fruit health states (0% overlap). Diseased fruits harboured exclusively Enterobacterales species, while healthy fruits harboured exclusively endophytic *Bacillus* species. The findings visually contrast the restricted bacterial community associated with healthy fruits against the broader diversity recovered from diseased fruits, providing a categorical overview of isolate occurrence across fruit conditions.

Data are based on 120 healthy and 120 diseased fruits, with all isolation and plating steps performed in triplicate per fruit‑health category. The absence of Enterobacterales from healthy‑fruit isolations and the restriction of *Bacillus* spp. to healthy fruits were observed consistently across all replicates and were statistically supported by Fisher’s exact test (*p < 0.001*).

**Fig. 3:**
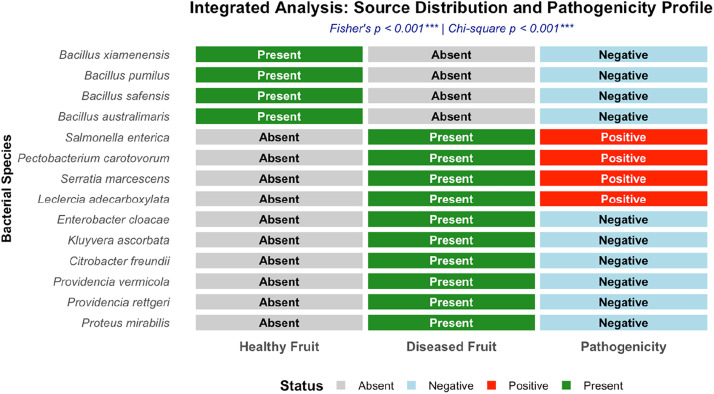
The integrated distribution and pathogenicity potential of bacterial isolates obtained from tomato fruits.

The integrated distribution and pathogenicity potential of bacterial isolates obtained from healthy and soft-rotten tomato fruits is shown in Fig. 3. This finding describes the link between each bacterial species to their source(s) of isolation (healthy or diseased fruits or both) and their observed preliminary pathogenicity in planta. All isolates recovered from healthy tomato fruits were identified as *Bacillus* species and showed no lesion development during pathogenicity screening or assays. In contrast, a subset of isolates recovered from diseased fruits exhibited pathogenic behaviour during tomato fruit inoculation. This integrated visualization enables cross-referencing of ecological origin and experimental pathogenicity outcomes without inferring causality. The findings clearly separate all non‑pathogenic *Bacillus* endophytes and saprophytic Enterobacterales from the four pathogenic species (*S. enterica, P. carotovorum, S. marcescens,* L. *adecarboxylata*), which are highlighted as soft-rot pathogens. Statistical analysis also indicated a highly significant association between both source distribution and pathogenic potential (Fisher's exact test, *p < 0.001*; Chi-square test, *p < 0.001*), indicating complete ecological and functional separation.

**Fig. 4:**
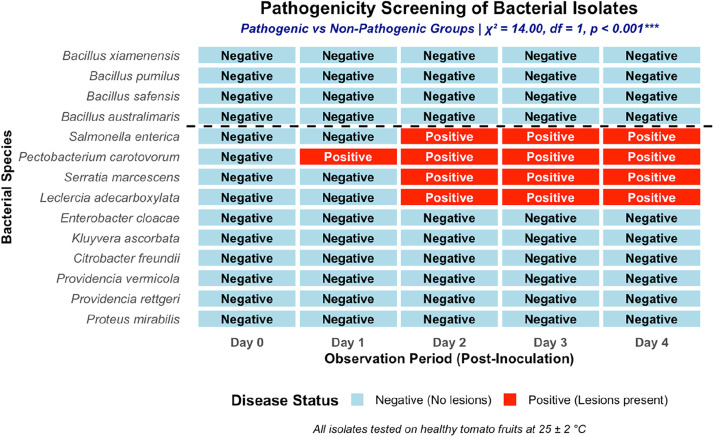
In planta pathogenicity screening of bacterial isolates after 5 days of inoculation.

A time‑resolved heat map of disease status (negative vs positive) for each isolate over four days after inoculation on healthy tomato fruits is shown in Fig. 4. The findings (Fig. 4) summarize the outcomes of *in planta* pathogenicity screening conducted on fresh tomato fruits over a five-day period following bacterial inoculation. Lesion development was first observed for *P. carotovorum* within 24 h post inoculation (Day 1). Other isolates exhibiting pathogenic responses, including *S. enterica, S. marcescens*, and L. *adecarboxylata*, developed visible lesions from Day 2 onward. In contrast, all *Bacillus* isolates and several Enterobacterales-associated species showed no visible disease symptoms throughout the monitoring period. At the final observation point, pathogenicity outcomes were recorded as a binary response based on lesion presence or absence. Chi-square analysis showed a highly significant association between isolate identity and lesion development at Day 4 (χ² = 14.00, df = 1, *p < 0.001*), with complete separation between pathogenic and non-pathogenic groups in this dataset. The classification of pathogenic versus non-pathogenic isolates remained consistent from Day 2 onwards, with four species consistently producing lesions and ten remaining non-pathogenic throughout the monitoring period. At the final observation point, pathogenicity outcomes were recorded as a binary response based on lesion presence or absence.

**Fig. 5:**
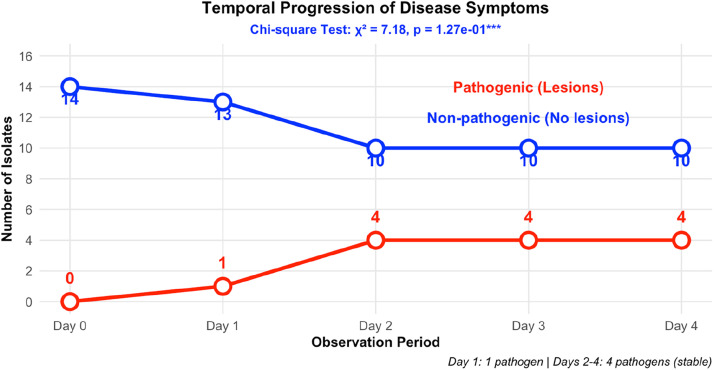
Temporal progression of disease symptoms during preliminary pathogenicity screening.

The temporal progression of disease symptoms during preliminary pathogenicity screening is shown in Fig. 5. The line plot shows that the number of pathogenic isolates increased from zero at day 0 to four by day 2 and then remained stable, while the count of non‑pathogenic isolates decreased correspondingly from 14 to 10, an indication that only a small, stable subset of species is responsible for soft‑rot development. The number of pathogenic isolates increased from zero at Day 0 to one at Day 1 (*P. carotovorum*) and stabilized at four by Day 2 (*S. enterica, P. carotovorum, S. marcescens,* L. *adecarboxylata*), remaining constant through Days 3 and 4 (χ² = 7.18, df = 4, *p* = 0.127). The consistent classification of four species as pathogenic from Day 2 onwards demonstrates the reproducibility of the pathogenicity screening assay. At day 1, lesion development was observed only for *P. carotovorum*. From day 2 onward, lesion development was recorded for *S. enterica, P. carotovorum, S. marcescens, and* L. *adecarboxylata*, with no additional changes observed through days 3 and 4. All remaining isolates consistently showed negative outcomes throughout the monitoring period. This Figure. provides time-resolved data on disease onset and persistence.

**Fig. 6:**
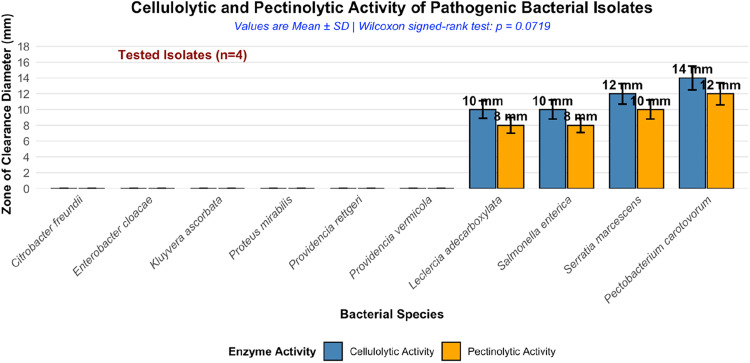
Cellulolytic and pectinolytic activity of bacterial isolates.

The cellulolytic and pectinolytic activity (phenotypic plant pathogenic virulence) of bacterial isolates from tomato fruits is shown in Fig. 6. The qualitative screening for extracellular cellulolytic and pectinolytic activities documents that detectable enzyme activity was restricted to isolates recovered from diseased tomato fruits. Saprophytic Enterobacterales showed no clear zones on cellulose- or pectin-containing media, indicating the absence of detectable hydrolytic activity under the assay conditions. In contrast, isolates that produced lesions *in planta* exhibited visible substrate degradation, with *P. carotovorum* displaying the largest clear zones, followed by *S. marcescens, S. enterica*, and L. *adecarboxylata*. The Figure. documents qualitative differences in cell wall–degrading enzyme phenotypes among isolates without quantitative measurement of enzymatic rates.

## Limitations

The datasets presented in our study were generated from tomato fruits obtained from selected open markets within a single city and during a single sampling period; consequently, the observed bacterial diversity and ecological patterns may not fully reflect those in other geographical regions, production systems, or seasonal conditions. Additionally, only culturable bacteria recovered using the media and incubation conditions applied were characterized, and unculturable, fastidious, or low-abundance taxa were not captured, as no high-throughput amplicon or metagenomic sequencing approaches were employed. Additionally, reliance on 16S rRNA gene sequencing for bacterial identification is a limitation because, for certain bacterial isolates, genus-level classification and that of closely related species within the genera, such as Bacillus and Pectobacterium, may require MLST using genes such as *gyrB* and *rpoB* for definitive identification. Future investigations should employ multilocus approaches to enhance taxonomic resolution. The pathogenicity screening was conducted on wounded tomato fruits under controlled laboratory conditions, which may overestimate disease potential relative to natural infection routes occurring in the field or along postharvest value chains. Furthermore, cellulolytic and pectinolytic activities were assessed using qualitative plate-based assays, providing comparative indications of enzyme activity rather than quantitative or kinetic measurements, and other potential virulence traits, such as biofilm formation, toxin production, and stress tolerance, were not evaluated. Lastly, the number of isolates examined per species was limited, restricting assessment of intra-species variability in pathogenicity and enzyme production; therefore, the findings should be interpreted as indicative rather than exhaustive for each taxon.

## Related research article

Ogofure, A.G., Ologbosere, A.O. (2023). Microbiological and Proximate Properties of Healthy and Diseased/spoilt (Broken) Tomatoes (*Lycopersicum esculentum* L.) Sold in Open Markets in Benin City: Public Health Implications. *J. Mat. Environ. Sci.* 14(4), 395–409

## Ethics statements

This research involved the random collection of tomato fruits from open markets in Benin City, Nigeria. No endangered or protected plant species were involved.

## CRediT author statement

**Abraham Goodness Ogofure:** Methodology, Formal analysis, Resources, Data curation, Visualization, Writing - original draft, Writing - review & editing.

**Ezekiel Green:** Supervision, Conceptualization, Investigation, Resources, Project administration, Funding acquisition, Writing - review & editing.

**Etinosa O. Igbinosa:** Conceptualization, Formal analysis, Investigation, Resources, Project administration, Funding acquisition, Writing - review & editing

Supplementary material and/or additional information [OPTIONAL]

## Declaration of competing interest

The authors declare that they have no known competing financial interests or personal relationships that could have appeared to influence the work reported in this paper.

## Data Availability

Data will be made available on request.

## Appendix Supplementary materials

Image, application 1
