# Supplementary material for: A reproducible workflow for isolating and characterizing bacterial endophytes, pathogens, and saprophytic colonizers from tomato fruits
Source: MethodsX. 2026 Mar 27;16:103889. doi: 10.1016/j.mex.2026.103889 (PMC13068625; doi:10.1016/j.mex.2026.103889)
Supplement: Supplementary file 1 [file mmc1.docx]

**
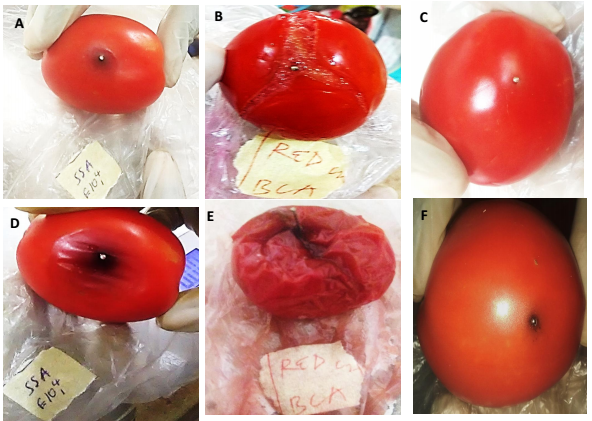
**

**Plate 1. Pathogenicity test of bacterial pathogens of tomato fruits A &D for *Salmonella* while B & E for *Serratia* C & F for control**

**
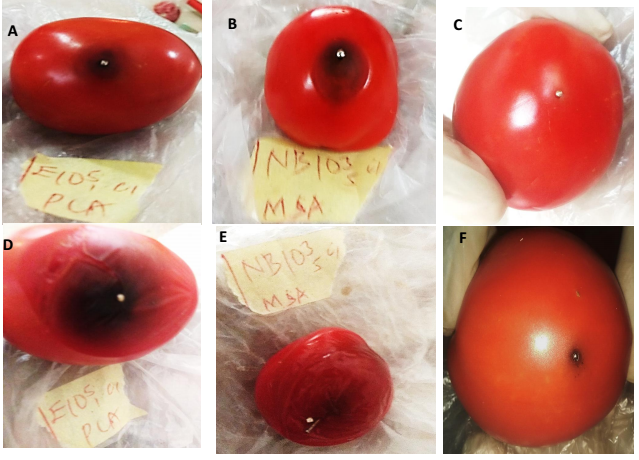
**

**Plate 2. Pathogenicity test of bacterial pathogens of tomato fruits A &D for *Leclercia* while B & E for *Pectobacterium,* C & F for control**
